# Induction of labor and postpartum blood loss

**DOI:** 10.1186/s12884-019-2410-8

**Published:** 2019-07-25

**Authors:** Romana Brun, Emilian Spoerri, Leonhard Schäffer, Roland Zimmermann, Christian Haslinger

**Affiliations:** 1 Division of Obstetrics, University Hospital Zurich, Frauenklinikstrasse 10, 8091 Zurich, Switzerland; 2 University of Zurich, Zurich, Switzerland; 3 Division of Obstetrics, Kantonsspital Baden, Baden, Switzerland

**Keywords:** Induction of labor, Spontaneous onset of labor, Postpartum hemorrhage, Postpartum blood loss, Delta hemoglobin

## Abstract

**Background:**

To analyze blood loss after delivery in women with induction of labor compared to women with spontaneous onset of labor.

**Methods:**

In this secondary analysis of a prospective cohort study investigating postpartum hemorrhage, 965 deliveries were analyzed including 380 women with induction of labor (39%) between 2015 and 2016. Primary outcome parameters were rate of postpartum hemorrhage, estimated blood loss and post-partum decrease in hemoglobin.

**Results:**

Rates of postpartum hemorrhage and estimated blood loss were not significantly different in women with induction of labor. Women with induction of labor had a significantly reduced decrease in hemoglobin after delivery. In the multivariate linear regression analysis, induction of labor remained associated with reduced decrease in hemoglobin. Secondary maternal and neonatal outcomes were unaffected.

**Conclusions:**

Induction of labor is not associated with increased blood loss after delivery and should not be regarded as a risk factor for postpartum hemorrhage.

## Background

Postpartum Hemorrhage (PPH) is defined by the WHO as blood loss of 500 ml or more within 24 h after birth [1]. Another, less commonly used definition distinguishes vaginal deliveries (blood loss > 500 ml) from cesarean sections (> 1000 ml) [2]. PPH affects approximately 2% of all women giving birth and remains a leading cause of maternal morbidity (blood transfusion, maternal admission to an intensive care unit [ICU], hysterectomy) and mortality. Maternal death due to PPH occurs in 1/1000 deliveries in developing countries and in 1/100000 deliveries in high-income countries such as the U.K. [3, 4].

Currently, average rates of induction of labor (IOL) based on international studies are approximately 20–25% of all pregnancies [5, 6]. Aside from medical reasons, IOL upon maternal request is a common procedure [7].

The risk for PPH following IOL has been published with partially conflicting results, even though many of the studies suggested that IOL would be associated with PPH. However, the majority of available studies were not designed to analyze the effect of IOL on blood loss following delivery [8–19] due to an imperfect analysis of other important risk factors for increased blood loss after delivery. Furthermore, none of the studies so far analyzed the correlation between IOL and linear variables of blood loss such as estimated blood loss (EBL) or post-partum decrease in hemoglobin (ΔHb), even less so in a multivariate analysis.

The objective of our study was to analyze blood loss in women with IOL compared to women with spontaneous onset of labor (SOL). Blood loss was assessed by rate of PPH, EBL (mL), and ΔHb (in g/l). Secondary outcome parameters included maternal and neonatal outcome after IOL.

## Methods

This was a secondary subgroup analysis of a prospective cohort study investigating risk factors for PPH at University Hospital Zurich, a tertiary care perinatal center, between October 2015 and November 2016 (PPH-study, ClinicalTrials.govID: NCT02604602). The study has ethical approval according to the ethics review board of Zurich (reference number KEK-ZH 2015–0011, date of approval: 4.9.2015).

Data were collected prospectively and recorded immediately after the delivery by the midwife and the attending physician in our electronic database which contains all the patients’ diagnoses and prospectively collected clinical data on the course of the pregnancy, delivery as well as maternal and infant outcome. A staff of three trained research personnel extracted the collected data prospectively into the study database using three clinical informatics systems: IntelliSpace Perinatal (OB TraceVue [OBTV], https://www.usa.philips.com), Perinat, and KISIM (both University Hospital Zurich products). The data was double-checked by the principal investigators and in case of dubious values the patient’s charts were studied again.

Inclusion criteria were planned vaginal delivery, written patient consent, singleton gestation, > 36 + 0 weeks of gestation and vertex position. Exclusion criteria were breech position, elective cesarean section, placenta previa, multiple fetus pregnancies and known coagulation disorders. Eligible patients were included consecutively into the prospective study. In total, 965 out of the 1500 patients of the primary study fulfilled the inclusion criteria, including 380 women with IOL (39.4%) and 585 (60.6%) women with SOL. Being a secondary subgroup analysis, a sample size calculation was not performed.

Primary outcomes were the rate of PPH defined as blood loss of ≥500 ml after spontaneous delivery and ≥ 1000 ml after cesarean section within 24 h of delivery, estimated blood loss (EBL), ΔHb and rate of ΔHb ≥ 30 g/l. ΔHb ≥30 g/l was arbitrarily considered as clinically significant. In spontaneous deliveries, EBL is measured with a quantitative method in our institution. Immediately after delivery of the baby, a new pad was placed underneath the woman’s pelvis. In case of suspected increased bleeding, these pads were weighed on a neonatal scale which was present in every delivery room. If weighed blood loss exceeded 300 ml, a collector bag with a quantitative scale was installed under the woman’s pelvis. Blood loss was documented regularly until the removal of the collector bag, which was performed after successful treatment of PPH and normalized bleeding intensity. The overall EBL within 24 h after delivery was modified if a possible increased blood loss after removal of the collector bag occurred by weighing these pads again. This method of blood loss measurement enabled reliable blood loss estimation, as has been shown previously [20]. In our institution, determination of Hb level is performed routinely prepartum as well as 24 to 48 h postpartum for all parturient women. Every value of hemoglobin was inserted and interpreted individually in our prospective database. In women with increased blood loss, several blood tests are likely to be performed and trained research personnel are essential to choose the appropriate hemoglobin value since hemoglobin concentration immediately after bleeding will be measured falsely high due to still outstanding auto-transfusion and hemoglobin concentration immediately after intensive fluid replacement therapy will be quantified falsely low [20]. Usually the clinically most reasonable hemoglobin value in women with increased bleeding represents the level 48 h postpartum. Necessity of blood transfusion was evaluated as well. In case of blood transfusion, the hemoglobin level before the transfusion was chosen.

In case of discrepancy between observed clinical course and extracted EBL or ΔHb, the patient’s chart was checked together with responsible physicians and midwives.

Secondary outcome variables included mode of delivery (spontaneous vaginal delivery, operative vaginal delivery, and unplanned cesarean section), length of hospital stay, need for surgical management due to PPH (such as B-Lynch sutures or hysterectomy), and neonatal outcome parameters (admission to neonatal care unit, arterial umbilical cord pH, 5′ and 10′ Apgar score, shoulder dystocia, and meconium staining of the amniotic fluid).

Furthermore, data on the following obstetric parameters were collected: IOL, method and duration of induction, gestational age at delivery, parity, maternal age at delivery, body mass index (BMI), duration of the first and second stage of labor, type of anesthesia during labor, uterus atony, retention of placenta or placental tissue, placental abruption, morbidly adherent placenta (placenta accreta, increta, and percreta), admission to intensive care unit, uterine rupture, amnion infection, anal sphincter tear, and infant birthweight.

Methods for IOL were oxytocin, misoprostol or both (misoprostol followed by oxytocin). Misoprostol induction was conducted with 25 micrograms of vaginal misoprostol repeated after 4 h if no contractions were noted up to a daily maximum dosage of 150 micrograms [21]. Oxytocin infusion via an infusion pump was initiated with 0.002 IU/minute and increased until adequate uterine activity (3–4 contractions/10 min) was obtained. Women with previous cesarean sections did not receive misoprostol.

Baseline as well as maternal and neonatal outcome parameters were compared between women with IOL and SOL. Differences between groups were analyzed with the Mann-Whitney U test for continuous variables and the Chi- square test or the Fisher’s exact test for categorical variables as appropriate. A multivariate linear regression analysis was performed to evaluate the association of the following variables on ΔHb: IOL, prolonged duration of induction (> 48 h), maternal BMI, gestational age, maternal age, repeat cesarean section and unplanned cesarean section, multiparity, duration of the second stage of labor > 2 h, retention of placenta or placental tissue, fetal weight, placental abruption, uterus atony.

All statistical analyses were performed using IBM SPSS Statistics, version 22.0 (Armonk, NY: IBM Corp.). Due to multiple testing and in order to avoid the multiple comparisons problem which increases the probability of a false positive finding, level of statistical significance was set at *p* < 0.01.

## Results

Baseline characteristics showed significant difference regarding maternal BMI, gestational age and birthweight (Table 1).

**Table 1:** Baseline Characteristics

Characteristics	IOL (*n* = 380)	SOL (*n* = 585)	*p*
Multiparity	155 (40.8%)	270 (46.2%)	0.101^a^
BMI (kg/m^2^)	24.9 ± 4.8	23.8 ± 4.5	< 0.001^b^
Age in years	32.2 ± 5	32.4 ± 4.8	0.51^b^
Gestational age in days	280 ± 9.1	277 ± 8.4	< 0.001^b^
Birthweight in g	3440 (3130–3739)	3340 (3065–3613)	< 0.01^b^

Data presented as n (%), mean ± SD or median (25th percentile – 75th percentile) as appropriate. *n* number, *SD* standard deviation, ^a^: Chi square Test, ^b^: Mann Whitney U Test

As method of induction, 138 (36.3%) women received oxytocin, 199 (52.4%) misoprostol and 43 (11.3%) misoprostol followed by oxytocin.

Incidence of PPH did not differ between women with IOL compared to women with SOL (24.7% vs. 21.2%, *p* = 0.20). This was also the case when using the PPH-definition proposed by the WHO (PPH defined as a blood loss of 500 ml or more within 24 h after birth, irrespective of the mode of delivery) [1]. Likewise, rates of EBL ≥1000 ml or ≥ 1500 ml did not differ between groups. The estimated blood loss was comparable in the IOL group (median 400 [300–600] ml vs 400 [300–500] ml, *p* = 0.03) while ΔHb was significantly lower in women with IOL (median 13 [5–21] g/l vs 16 [9.0–24] g/l, *p* < 0.01). ΔHb > 30 g/l was not significantly different in study group vs. controls (12.9% vs. 13.8%, *p* = 0.67).

Rates of vaginal-operative delivery and unplanned cesarean section as well as length of hospital stay were not different.

All secondary neonatal outcome parameters were comparable between both groups. Maternal and neonatal outcome data is shown in Table 2. None of our patients needed surgery to treat PPH (B-Lynch sutures or hysterectomy).

**Table 2:** Maternal and neonatal outcome

Blood loss parameters	Women with IOL (*n* = 380)	Women with SOL (*n* = 585)	*p*
Estimated blood loss (mL)	400 (300–600)	400 (300–500)	0.03 ^a^
Estimated blood loss ≥500 mL	152 (40%)	195 (33.3%)	0.03 ^b^
Estimated blood loss ≥1000 mL	24 (6.3%)	37 (6.3%)	1.00 ^b^
Estimated blood loss ≥1500 mL	9 (2.4%)	10 (1.7%)	0.47 ^b^
Postpartum Hemorrhage	94 (24.7%)	124 (21.2%)	0.20 ^b^
Delta hemoglobin (g/L)	13 (5–21)	16 (9.0–24.0)	< 0.01 ^a^
Delta hemoglobin ≥30 g/L	49 (12.9%)	81 (13.8%)	0.67 ^b^
Blood transfusion	1 (0.3%)	0 (0.0%)	0.39 ^c^
Secondary maternal outcome			
Vaginal delivery	238 (62.6%)	390 (66.7%)	0.20 ^b^
Operative vaginal delivery	51 (13.4%)	72 (12.3%)	0.61 ^b^
Second stage of labor > 120 min	93 (30.6%)	132 (26.7%)	0.23 ^b^
Unplanned cesarean section	91 (24%)	123 (21.0%)	0.29 ^b^
Maternal ICU admission	2 (0.5%)	0 (0.0%)	0.15 ^c^
Uterine atony	28 (7.4%)	25 (4.3%)	0.04 ^b^
Retained placenta	10 (2.6%)	16 (2.7%)	0.92 ^b^
Retained placental tissue	15 (3.9%)	13 (2.2%)	0.12 ^b^
Placental abruption	1 (0.3%)	5 (0.9%)	0.41 ^c^
Uterine rupture	1 (0.3%)	2 (0.3%)	1.0 ^c^
Length of hospital stay after delivery	4 (3–4)	4 (3–4)	0.07 ^a^
Anal sphincter tear	6 (1.6%)	4 (0.7%)	0.20 ^c^
Secondary Neonatal outcome			
Chorioamnionitis	5 (1.3%)	3 (0.5%)	0.28 ^c^
Shoulder dystocia	7 (1.8%)	10 (1.7%)	0.88 ^b^
Meconium staining	70 (18.4%)	107 (18.3%)	0.96 ^b^
Umbilical arterial pH < 7.1	5 (1.3%)	11 (1.9%)	0.50 ^b^
5′ Apgar score < 7	4 (1.1%)	6 (1.0%)	1.0 ^c^
10′ Apgar score < 7	1 (0.3%)	0 (0%)	0.39 ^c^
Admission to neonatal care unit	23 (6.1%)	16 (2.7%)	0.11 ^b^

*IOL* induction of labor, *SOL* spontaneous onset of labor, Postpartum Hemorrhage: blood loss of ≥500 ml after spontaneous delivery or ≥ 1000 ml after cesarean section within 24 h of delivery

Data presented as n (%), or median (25th percentile – 75th percentile) as appropriate. *n* number, *SD* standard deviation; ^a^: Mann Whitney U- Test, ^b^ Chi Square Test, ^c^: Fisher’s Exact test

In a multivariable linear regression analysis, IOL was independently associated with reduced ΔHb (− 3.4 g/l [CI 95% -4.98 to − 1.79], *p* < 0.001). Other factors that had a statistically significant impact on ΔHb were multiparity, second stage of labor > 120 min, uterine atony, retention of placenta and placental tissue (Table 3). Prolonged IOL (> 48 h) was not associated with increased blood loss in the multivariable regression analysis.

**Table 3:** Multivariable linear regression analysis for postpartum decrease in hemoglobin (ΔHb)

	Influence on ΔHb (95% CI) in g/L	Correlation coefficient	*p*
Induction of labor	−3.9 (−5.5 to −2.4)	−0.15	< 0.001
BMI (kg/m^2^)	0.1 (−0.0 to 0.3)	0.05	0.09
Gestational age in days	0.0 (− 0.1 to 0.1)	0.01	0.83
Maternal age in years	−0.1 (− 0.2 to 0.1)	−0.33	0.26
Multiparity	−6.1 (−7.7 to −4.4)	−0.23	< 0.001
Second stage of labor > 120 min	7.4 (5.6 to 9.3)	0.26	< 0.001
Retained placenta	15.7 (11.5 to19.9)	0.22	< 0.001
Retained placental tissue	11.9 (7.8 to 16.0	0.17	< 0.001
Birthweight in g	0.0 (−0.0 to 0.0)	−0.02	0.66
Placental abruption	6.4 (−4.1 to 16.9)	0.04	0.24
Repeat cesarean section	7.8 (−0.8 to 16.4)	0.06	0.07
Unplanned cesarean section	2.7 (−0.6 to 6.1)	0.05	0.11
Uterine Atony	14.7 (11.5 to 17.9)	0.27	< 0.001
Induction of labor > 48 h	2.1 (−2.5 to 6.7)	0.03	0.36

## Discussion

This secondary analysis of a prospective study tested for the impact of IOL on postpartum blood loss. To our best knowledge, this is the first study to analyze prospectively collected data about blood loss after IOL as primary outcome parameter with adjustment for important risk factors of PPH.

We showed that IOL is not associated with increased blood loss after delivery. IOL was even associated with a slight, however statistically significant, reduced decrease in hemoglobin after delivery compared to SOL. This finding remained statistically significant in the multivariable linear regression analysis. According to this only slightly reduced decrease in hemoglobin after IOL, a clinical relevant loss of hemoglobin defined as ΔHb ≥30 g/l was not different in women with IOL as compared to women with SOL.

Our findings are in accordance with a recent Cochrane Review [22], which assessed the outcome of IOL in women at or beyond term (i.e., from 37 completed weeks of pregnancy). With regard to PPH, four published randomized controlled trials and one abstract were included with no evidence of a difference between IOL and SOL (RR 1.09 95%CI 0.92 to 1.30, low-quality evidence). However, all of these trials were primarily designed to evaluate the mode of delivery (cesarean rate) [23–25] or neonatal outcome [26] but not postpartum blood loss. Hence, there was no information about important causes for PPH such as uterine atony, retained placenta, increased bleeding from lacerations or coagulopathy. Furthermore, analysis of decrease in hemoglobin after delivery as an objective parameter for blood loss was not performed in any of these studies.

In contrast to the above mentioned trials and to our findings, eight of nine retrospective studies [8–10, 12, 13, 15–17, 27] that examined IOL as a possible risk factor for PPH, found IOL to be associated with the occurrence of PPH [8–10, 13, 15–17, 27].

Reported odds ratios for blood loss > 500 mL after IOL ranged from 1.4 to 4.1 [8, 9, 15, 27] and those for blood loss > 1500 mL were 1.6 [17]. However, there are important methodological issues to discuss in these analyses. In seven of these nine studies, no adequate multivariate analysis for the above mentioned acknowledged risk factors for PPH was performed [8–10, 12, 13, 15, 16]. Furthermore, decrease in hemoglobin after delivery was analyzed in only one study [13], in which no multivariate analysis was performed. Finally and most importantly, five studies [8, 15–17, 27] were not designed to analyze IOL and its impact on PPH, but rather studied women with PPH and retrospectively looked at possible risk factors for PPH. Albeit these studies are undoubtedly of importance to anticipate prospective studies, they are of low value to discuss a single risk factor such as IOL alone as comparison of patients subgroups (with or without IOL) isn’t feasible.

A secondary analysis of a large French trial, which analyzed the effect of a multifaceted intervention on the reduction of severe PPH, assessed the association between IOL and PPH in low-risk parturients [19]. It suggested that only women with IOL following a standard indication were at higher risk of PPH (adjusted OR 1.22, 95%CI 1.04–1.42), however not of severe PPH. Besides having substantial strengths such as inclusion of a large number of women (*n* = 6′621) and detailed information about the mode of IOL as well as the cervical status, the study showed limitations that might have contributed to the heterogeneous results. For instance, the main obstetrical reasons for increased postpartum blood loss, such as uterine atony and retained placenta, were not included in the analysis and no data about blood loss (in ml) or loss of hemoglobin (in g/l) was reported.

To our knowledge, only one prospective study has evaluated risk factors for PPH as a primary outcome among approximately 11′300 South-American women, showing IOL be one of them [18]. In this study by Sosa et al., IOL was associated with severe PPH defined as blood loss > 1000 mL (OR 2.0) but, interestingly, not with PPH defined as blood loss > 500 mL. However, as IOL was not the main focus of the study, no information was given about patient characteristics in the IOL and SOL groups. In addition, analysis of ΔHb was not performed.

In conformity with recent, partly randomized controlled studies [5, 11, 23–26] and in contrast to some retrospective studies [9, 10, 12, 28], IOL was not associated with an increased risk for unplanned cesarean section or operative vaginal delivery in our study. The apparently high incidence of IOL in our study population (39%) is explained by the study design, which excluded all patients with preterm deliveries before 36 weeks of gestation. Unfortunately, our study did not provide information about the medical indication for IOL.

Multiparity was associated with a reduced decrease in hemoglobin. Most of our multiparous patients had their second or third delivery (67 and 24% respectively) leaving only 11 patients with grand multiparity which is at higher risk for complications. We think that in these multiparous patients, blood loss is reduced due to faster deliveries with a shorter second stage of laborEven though our study is neither conducted nor designed for analyzing other secondary outcomes such as length of hospital stay, anal sphincter tears or neonatal outcome parameters, we observed no difference between the IOL and SOL groups.

A main strength of our study was its design as a secondary subgroup analysis of a prospective PPH study, thus guaranteeing thorough assessment of blood loss parameters and risk factors. The calculation of ΔHb by measurement before and after delivery poses an objective parameter to examine blood loss during birth. Interpretation of hemoglobin values for each study patient by trained research personnel, as described above, is of absolute importance, especially in women with increased blood loss requiring several postpartum blood specimens. In vaginal deliveries, estimated blood loss was determined by our plastic bag system with a quantitative scale which enables a reasonably precise measurement of blood loss. It is generally accepted that without a quantitative measurement method, blood loss is prone to underestimation [29, 30].

Limitations of our study are the non-randomized study design and the missing sample size calculation which is impossible in secondary analysis. Hence, our results need to be interpreted with caution. However, in contrast to the existing randomized controlled trials, our study design was developed to assess postpartum blood loss with a focus on reliable analysis of blood loss including decrease in hemoglobin after delivery. Another limitation is missing data about history of PPH, use of oxytocin for labor augmentation during delivery and previous cesarean sections in our patients. However, uterine rupture as the main reason for increased blood loss in patients with previous cesarean section was included in the analysis and did not show any difference between groups.

## Conclusions

IOL is not associated with increased blood loss after delivery. In fact, women with IOL show a statistically significant reduced decrease in hemoglobin after delivery, which is however clinically not relevant. Hence, IOL is a safe procedure regarding PPH and cannot be regarded as a risk factor for the incidence of PPH or increased blood loss after delivery.

## Data Availability

The datasets used and/or analyzed during the current study are available from the corresponding author on reasonable request.

## Abbreviations

EBL: Estimated blood loss
ICU: Intensive care unit
IOL: Induction of labor
PPH: Postpartum hemorrhage
SOL: Spontaneous onset of labor
ΔHb: Delta hemoglobin (post-partum decrease in hemoglobin)

## References

[1] WHO Guidelines Review Committee. WHO Guidelines Approved by the Guidelines Review Committee. In: WHO Recommendations for the Prevention and Treatment of Postpartum Haemorrhage. edn. Geneva: World Health OrganizationWorld Health Organization; 2012.

[2] Solomon C, Collis RE, Collins PW (2012). Haemostatic monitoring during postpartum haemorrhage and implications for management. Br J Anaesth.

[3] Mousa HA, Blum J, Abou El Senoun G, Shakur H, Alfirevic Z. Treatment for primary postpartum haemorrhage. Cochrane Database Syst Rev. 2014(2). 10.1002/14651858.CD003249.pub3. 10.1002/14651858.CD003249.pub3 PMC6483801 24523225

[4] Oyelese Y, Ananth CV (2010). Postpartum hemorrhage: epidemiology, risk factors, and causes. Clin Obstet Gynecol.

[5] Nicholson JM, Kellar LC, Henning GF, Waheed A, Colon-Gonzalez M, Ural S (2015). The association between the regular use of preventive labour induction and improved term birth outcomes: findings of a systematic review and meta-analysis. BJOG.

[6] Mozurkewich E, Chilimigras J, Koepke E, Keeton K, King VJ (2009). Indications for induction of labour: a best-evidence review. BJOG.

[7] Coulm B, Blondel B, Alexander S, Boulvain M, Le Ray C (2016). Elective induction of labour and maternal request: a national population-based study. BJOG.

[8] Hiersch L, Bergel-Bson R, Asher D, Aviram A, Gabby-Benziv R, Yogev Y, Ashwal E (2017). Risk factors for post-partum hemorrhage following vacuum assisted vaginal delivery. Arch Gynecol Obstet.

[9] Selo-Ojeme D, Rogers C, Mohanty A, Zaidi N, Villar R, Shangaris P (2011). Is induced labour in the nullipara associated with more maternal and perinatal morbidity?. Arch Gynecol Obstet.

[10] Vardo JH, Thornburg LL, Glantz JC (2011). Maternal and neonatal morbidity among nulliparous women undergoing elective induction of labor. J Reprod Med.

[11] Zhang L, Zhang H, Zhang J, Zhang JW, Ye JF, Branch DW (2016). Preventive induction of labor for non-urgent indications at term and maternal and neonatal outcomes. Reprod Health.

[12] Bailit JL, Grobman W, Zhao Y, Wapner RJ, Reddy UM, Varner MW, Leveno KJ, Caritis SN, Iams JD, Tita AT (2015). Nonmedically indicated induction vs expectant treatment in term nulliparous women. Am J Obstet Gynecol.

[13] Phillip H, Fletcher H, Reid M (2004). The impact of induced labour on postpartum blood loss. J Obstet Gynaecol.

[14] ElSedeek M, Awad EE, ElSebaey SM (2009). Evaluation of postpartum blood loss after misoprostol-induced labour. BJOG.

[15] Rossen J, Okland I, Nilsen OB, Eggebo TM (2010). Is there an increase of postpartum hemorrhage, and is severe hemorrhage associated with more frequent use of obstetric interventions?. Acta Obstet Gynecol Scand.

[16] Bais JM, Eskes M, Pel M, Bonsel GJ, Bleker OP (2004). Postpartum haemorrhage in nulliparous women: incidence and risk factors in low and high risk women. A Dutch population-based cohort study on standard (> or = 500 ml) and severe (> or = 1000 ml) postpartum haemorrhage. Eur J Obstet Gynecol Reprod Biol.

[17] Al-Zirqi I, Vangen S, Forsen L, Stray-Pedersen B (2008). Prevalence and risk factors of severe obstetric haemorrhage. BJOG.

[18] Sosa CG, Althabe F, Belizan JM, Buekens P (2009). Risk factors for postpartum hemorrhage in vaginal deliveries in a Latin-American population. Obstet Gynecol.

[19] Khireddine I, Le Ray C, Dupont C, Rudigoz RC, Bouvier-Colle MH, Deneux-Tharaux C (2013). Induction of labor and risk of postpartum hemorrhage in low risk parturients. PLoS One.

[20] Kahr MK, Brun R, Zimmermann R, Franke D, Haslinger C (2018). Validation of a quantitative system for real-time measurement of postpartum blood loss. Arch Gynecol Obstet.

[21] American College of Obstetricians and Gynecologists. ACOG Practice Bulletin No. 107: Induction of labor. Obstet Gynecol. 2009;114(2 Pt 1):386–97. 10.1097/AOG.0b013e3181b48ef5 19623003

[22] Middleton P, Shepherd E, Crowther CA (2018). Induction of labour for improving birth outcomes for women at or beyond term. Cochrane Database Syst Rev.

[23] Walker KF, Bugg GJ, Macpherson M, McCormick C, Grace N, Wildsmith C, Bradshaw L, Smith GC, Thornton JG (2016). Randomized trial of labor induction in women 35 years of age or older. N Engl J Med.

[24] Chanrachakul B, Herabutya Y (2003). Postterm with favorable cervix: is induction necessary?. Eur J Obstet Gynecol Reprod Biol.

[25] Brane E, Olsson A, Andolf E (2014). A randomized controlled trial on early induction compared to expectant management of nulliparous women with prolonged latent phases. Acta Obstet Gynecol Scand.

[26] Heimstad R, Skogvoll E, Mattsson LA, Johansen OJ, Eik-Nes SH, Salvesen KA (2007). Induction of labor or serial antenatal fetal monitoring in postterm pregnancy: a randomized controlled trial. Obstet Gynecol.

[27] Sheiner E, Sarid L, Levy A, Seidman DS, Hallak M (2005). Obstetric risk factors and outcome of pregnancies complicated with early postpartum hemorrhage: a population-based study. J Matern Fetal Neonatal Med.

[28] Luthy DA, Malmgren JA, Zingheim RW (2004). Cesarean delivery after elective induction in nulliparous women: the physician effect. Am J Obstet Gynecol.

[29] Dildy GA, Paine AR, George NC, Velasco C (2004). Estimating blood loss: can teaching significantly improve visual estimation?. Obstet Gynecol.

[30] Yoong W, Karavolos S, Damodaram M, Madgwick K, Milestone N, Al-Habib A, Fakokunde A, Okolo S (2010). Observer accuracy and reproducibility of visual estimation of blood loss in obstetrics: how accurate and consistent are health-care professionals?. Arch Gynecol Obstet.

